# Virtual reality for improving pain and pain-related symptoms in patients with advanced stage colorectal cancer: A pilot trial to test feasibility and acceptability

**DOI:** 10.1017/S1478951521002017

**Published:** 2022-08

**Authors:** Sarah A. Kelleher, Hannah M. Fisher, Joseph G. Winger, Shannon N. Miller, Grace H. Amaden, Tamara J. Somers, Luana Colloca, Hope E. Uronis, Francis J. Keefe

**Affiliations:** 1 Department of Psychiatry and Behavioral Sciences, Duke University Medical Center, Durham, NC;; 2 Department of Pain and Translational Symptom Science, School of Nursing and Department of Anesthesiology School of Medicine, University of Maryland, Baltimore, MD; 3 Department of Internal Medicine, Duke University Medical Center, Duke Cancer Institute, Durham, NC

**Keywords:** Advanced disease, Cancer pain, Colorectal cancer, Palliative care, Virtual reality

## Abstract

**Objective.:**

Virtual reality (VR) has the potential to improve pain and pain-related symptoms. We examined the feasibility, acceptability, safety, and impact of a 30-min virtual underwater/sea environment (VR Blue) for reducing pain and pain-related symptoms in advanced colorectal cancer patients. A qualitative exit interview was conducted to understand preferences, thoughts, and feelings about the VR session.

**Method.:**

Participants (*N* = 20) had stage IV colorectal cancer and moderate-to-severe pain. Participants completed a 30-min VR Blue session that visually and aurally immersed them in virtual ocean scenarios. Feasibility was assessed by accrual (*N* = 20), protocol adherence (≥80% completing VR Blue), and completed data (≥80% assessment completion). Acceptability was determined by patients reporting ≥80% intervention satisfaction. Safety was determined by ≥80% of patients completing the session without self-reported side effects. Measures of pain, tension, relaxation, stress, anxiety, and mood were collected before, during, and after the VR Blue session. A semi-structured qualitative interview was conducted after VR Blue to assess participants’ VR experiences.

**Results.:**

All participants (100%) completed the VR Blue session. There was 100% data collection at the pre- and post-assessments. Satisfaction with VR Blue was high *M* = 3.3 (SD = 0.4) (83%). No significant side effects were reported. Pain decreased by 59% (Pre-*M* = 3 [1]; Post-*M* = 1 [1]). Tension decreased by 74% (Pre-*M* = 30 [24]; Post-*M* = 8 [13]). Relaxation improved by 38% (Pre-*M* = 62 [21]); Post-*M* = 86 [17]). Stress decreased by 68% (Pre-*M* = 24 [24]; Post-*M* = 8 [14]). Anxiety decreased by 65% (Pre-*M* = 20 [23]; Post-*M* = 7 [13]). Mood improved by 70% (Pre-*M* = 13 [16]; Post-*M* = 4 [11]). Qualitative data suggested a positive response to the VR Blue protocol.

**Significance of results.:**

This work supports the feasibility, acceptability, and safety of VR Blue for advanced colorectal cancer patients. Participants showed significant pre-post improvement in pain and pain-related symptoms hinting to the potential feasibility of VR interventions in this population. Larger, randomized trials with a control condition are needed to examine the efficacy of VR-based interventions for patients with advanced colorectal cancer and pain.

## Introduction

Colorectal cancer is the fourth most commonly diagnosed malignancy in the USA (Siegel et al., 2018; Wolf et al., 2018). Advanced colorectal cancer is associated with elevated mortality and morbidity (Ryerson et al., 2016). Advanced colorectal cancer patients report high levels of pain and distress (i.e., stress and anxiety), and describe these symptoms to be especially burdensome (Pearce et al., 2008; Denlinger and Barsevick, 2009; Lowery et al., 2013). Persistent pain and distress are debilitating for patients with advanced colorectal cancer and negatively impact their quality of life (Denlinger and Barsevick, 2009; Gray et al., 2011).

Pain and distress among patients with advanced colorectal cancer are often not adequately treated. Traditional analgesic and other medication regimens for pain and pain-related symptoms do not provide full relief and patients report significant side effects (e.g., constipation, nausea, and sedation) that limit the use of analgesic medications (Miaskowski et al., 1994; Roeland et al., 2010). There is growing recognition of the role that non-pharmacological pain management strategies can play in treating pain and pain-related symptoms. Immersive virtual reality (VR) could represent a valuable treatment option for persistent pain in palliative care patients.

Recent evidence suggests VR interventions can lead to reductions in pain for patients with acute pain conditions (Hoffman et al., 2011; Keefe et al., 2012; Garrett et al., 2014; Morris et al., 2009). VR provides individuals with an immersive computer-generated environment that has the potential to reduce pain as well as tension and distress (Keefe et al., 2012). Despite the promise of VR as a non-pharmacological treatment for pain in the palliative cancer care setting, VR interventions have not yet been tested in advanced cancer patients with *persistent* pain. As outlined in the NIH Stage Model for Behavioral Intervention Development, determining whether an intervention is feasible and acceptable is crucial for successful intervention development (i.e., Stage 1) (Onken et al., 2014). Since a VR intervention has never been tested in advanced colorectal cancer samples, it is critical for early pilot work to first examine *a priori* feasibility, acceptability, and safety benchmarks. Additionally, pilot work should index primary outcomes with psychometrically sound measures that can detect minimally clinically important differences (MCID). The current pilot study will explore pre-post VR changes in primary outcome variables of pain, tension, distress (i.e., stress, anxiety, and mood), and relaxation. An MCID of 30% has been cited in the pain literature (Cleeland, 1991; Mease et al., 2011; Marcus et al., 2018) and will be used to suggest a significant change from pre- to post-VR Blue session. Data will provide preliminary support for conducting larger, randomized clinical trials.

Stage 1 pilot work is also an ideal opportunity to begin assessing mechanisms of change. Due to their late-stage disease and persistent pain, advanced colorectal cancer patients report negative pain-related cognitions (e.g., pain catastrophizing) and low confidence that they can control pain without medical intervention (e.g., self-efficacy for pain management). These cognitive pathways have been shown to be important for pain reduction within the context of cognitive-behavioral pain management protocols (Allen et al., 2019; White et al., 2019; Greenberg et al., 2020; Winger et al., 2020). VR may be particularly useful for advanced colorectal cancer patients endorsing persistent pain because it can impact cognitive pathways by decreasing pain catastrophizing and increasing pain self-efficacy to improve pain control and moderate pain signaling (Loreto-Quijada et al., 2014).

This pilot study extends preliminary research conducted by Colloca et al. (2020) that found exposure to a 30-min virtual underwater/sea environment (VR Blue) produced significant increases in pain tolerance for thermal pain stimuli compared to control conditions, along with improved mood and reduced anxiety and pain unpleasantness. This pilot study is the first to extend VR Blue to a clinical population and will assess feasibility by accrual, adherence to the protocol, and assessment completion. Acceptability of the proposed VR intervention will be assessed quantitatively via the self-report Client Satisfaction Questionnaire (CSQ) and qualitatively through a semi-structured interview. Given the novelty of this VR intervention for a vulnerable patient population, safety will be assessed to ensure the protocol is prudent for additional efficacy testing in advanced colorectal cancer samples. We collected data on primary outcome variables of pain, tension, distress, and relaxation pre-, mid-, and post-VR Blue. We also measured secondary outcomes of pain catastrophizing and self-efficacy for pain management to begin elucidating how changes in these constructs relate to changes in the primary outcome variables. Finally, we collected qualitative data following the VR Blue session to better understand participants’ preferences, thoughts, and feelings about the VR experience.

## Methods

### Participants

Participants (*N* = 20) were adult colorectal cancer patients with advanced disease (stage IV). Other eligibility criteria included: (1) age 18–85; (2) at least moderate pain (≥4 on 0–10 scale) on most days of the month for ≥3 months; (3) English-speaking; and (4) self-reported normal or corrected to normal vision and hearing. Patients were excluded if they had (1) a serious mental illness (e.g., schizophrenia) or medical condition (e.g., recent myocardial infarction) that would contraindicate safe participation; or (2) visual, hearing, or cognitive impairment that would interfere with engagement in the VR Blue session. All participants continued to receive their usual medical care and were not asked to change or decline any adjunct strategies for pain management. The Institutional Review Board at the Duke University Health System approved the study (Pro00103248) and all procedures complied with HIPAA guidelines. This study is registered on ClinicalTrials.gov (NCT04069702).

### Procedure

#### Recruitment

Participants were recruited from the Duke Cancer Institute in Durham, NC, USA. To assess eligibility, patient information was gathered from electronic medical records. After oncologist approval, potential participants were contacted via telephone and asked about their pain using the following questions: (1) “How would you rate your pain today on a scale from 0 (no pain) to 10 (pain as bad as you could imagine)?” (2) “Have you had pain on most days of the month for the past 3 months?” (3) “Do you take medication for your pain?” and (4) If you did not take medication for your pain, how would you rate your pain on a 0–10 scale?” To be eligible, patients were required to report current pain (without medication) at ≥4 on a 0–10 scale, and the pain must have been present on most days for the past 3 months. Vision and hearing, as well as cognitive status (6-item Mini-Mental State Exam; Folstein et al., 1975), were also assessed during the screening call. Patients who met the eligibility criteria and expressed interest were scheduled for an appointment to complete informed consent and the VR session.

#### Study design

Participants participated in a single, 30-min VR session of underwater/sea environments (VR Blue). Pre-, mid-, and post-VR Blue session assessments were completed online via REDCAP using an iPad, and measured self-reported pain, tension, distress (i.e., stress, anxiety, and mood), relaxation, pain catastrophizing, and self-efficacy for pain management. Participants also provided self-report demographic and medical data that were confirmed with electronic chart review. After the VR Blue session, participants completed a 10–15 min semi-structured exit interview to assess their preferences, thoughts, and feelings about the VR experience. Participants were compensated $40. Feasibility was assessed by (1) study accrual (*N* = 20); (2) protocol adherence (≥80% completing VR Blue); and (3) completed data (≥80% assessment completion). Acceptability was determined by ≥80% satisfaction on the CSQ at the post-VR Blue timepoint. Safety was determined by ≥80% of patients completing the session without significant side effects (e.g., motion sickness and dizziness).

#### VR session

**Fig 1.**
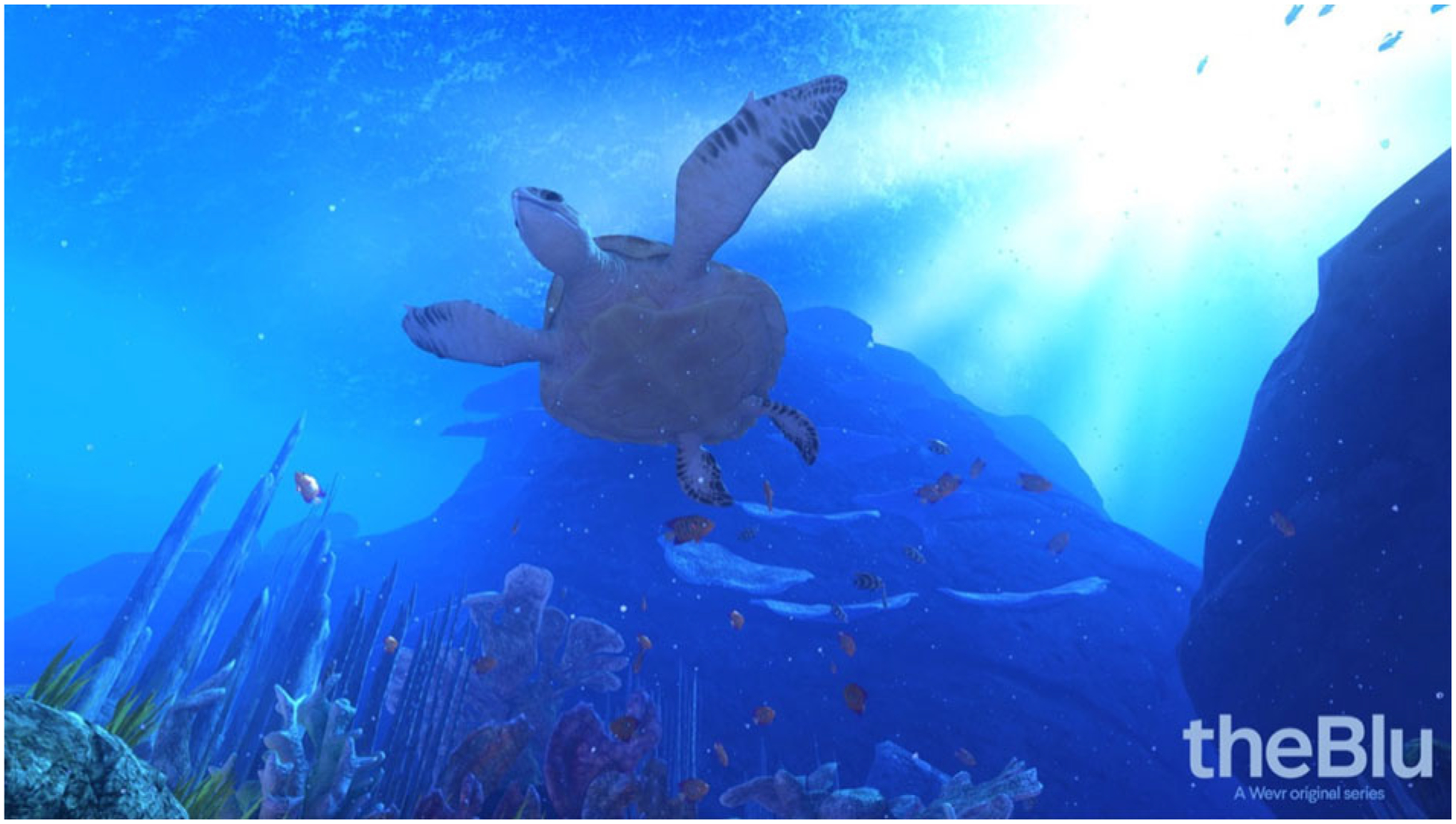
Image from VR Blue scene 1.

**Fig 2.**
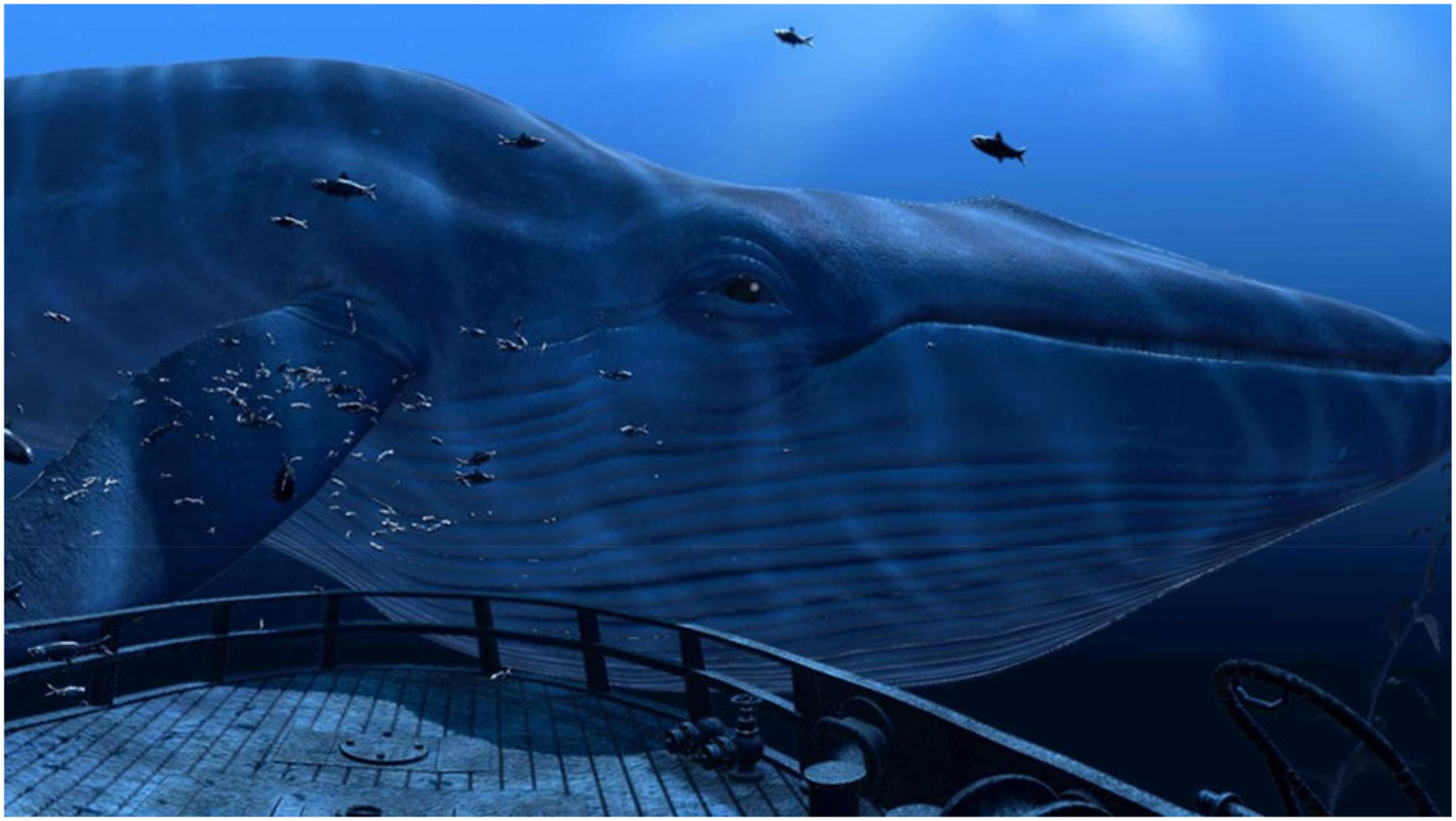
Image from VR Blue scene 2.

**Fig 3.**
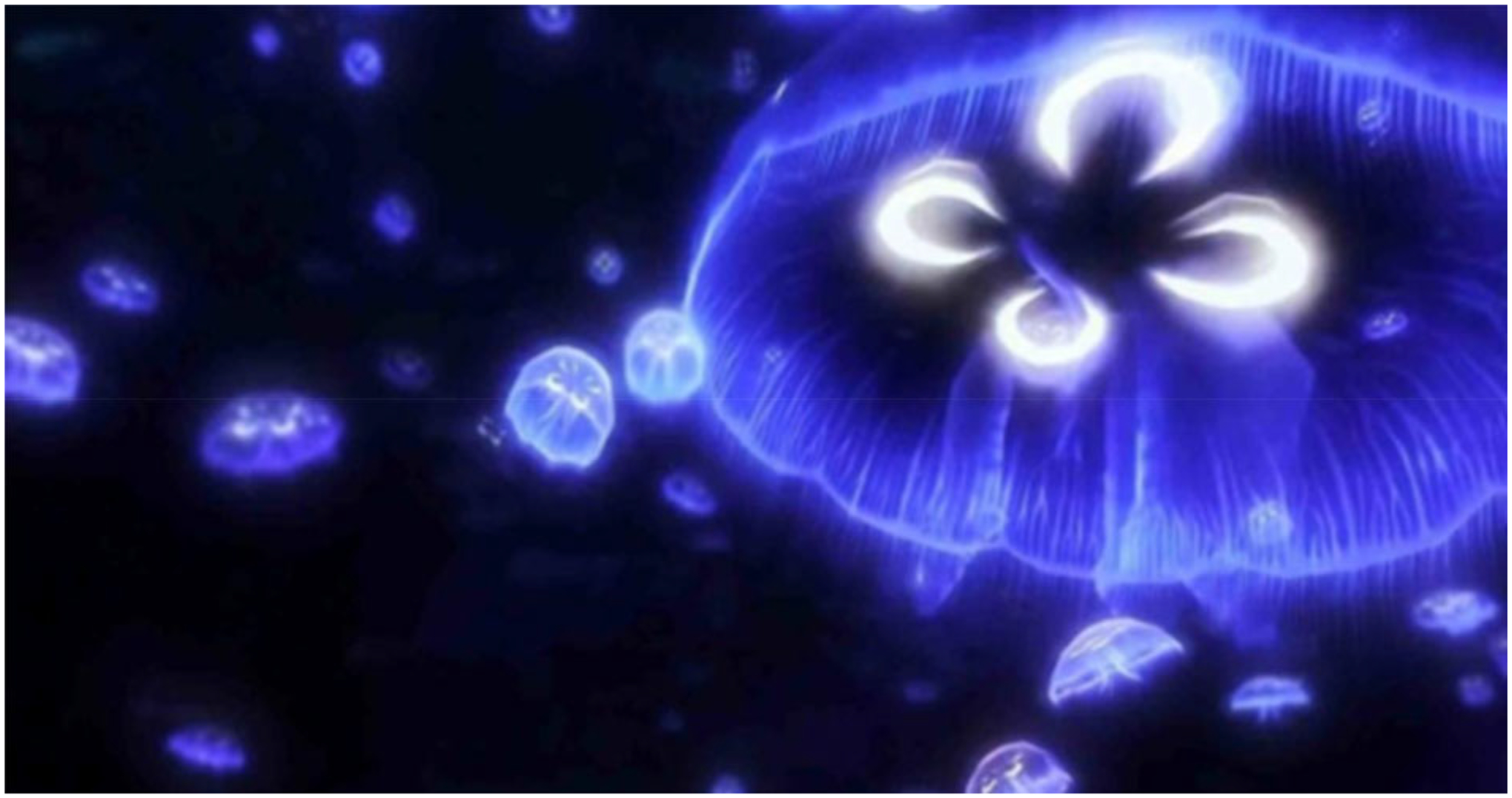
Image from VR Blue scene 3.

VR Blue was implemented using “theBlu” Season 1 (Wevr, 2021), an immersive computer-generated environment featuring calming scenic graphics of underwater/sea environments and relaxing nature music. The single 30-min VR Blue session allowed participants to experience the wonders of the ocean through exposure to three virtual environments: (1) a coral reef with turtles, fish, and other aquatic wildlife (Figure 1), (2) a shipwreck with a large whale (Figure 2), and (3) the deep sea (Figure 3). A study team member familiarized the participant with the VR glasses, controller, and screen, and guided the participant through a VR demonstration. Participants were oriented to the VR’s visual and auditory stimuli with an emphasis on becoming relaxed and fully immersed in the virtual environment. Following this orientation, participants completed the 30-min VR Blue session. VR has the potential to cause side effects known as “cybersickness” (i.e., dizziness, headache, and nausea) (Weech et al., 2019). The anticipated risks associated with cybersickness are comparable to the everyday use of computers (Keefe et al., 2012). Participants were asked to report any side effects immediately and had the option of discontinuing participation.

### Measures

#### Demographic variables

Demographic and medical variables were collected by self-report and electronic medical record review.

#### Feasibility

*A priori* feasibility benchmarks included: (1) reaching study accrual (*N* = 20) over 6 months; (2) ≥80% adherence to the protocol (i.e., degree of willingness/ability to complete the 30-min VR Blue session); and (3) ≥80% data collected at the study appointment.

#### Acceptability

The 10-item CSQ was used to assess acceptability post-VR Blue session (Attkisson and Zwick, 1982). Items were rated on a 4-point scale from 1 = low acceptability to 4 = high acceptability. Items are averaged to obtain a total score (Cronbach’s *α* = 0.84). Acceptability was demonstrated by participants reporting ≥80% satisfaction (*M* = 3.2/4.0) with the intervention.

#### Safety

Safety of the VR Blue session was assessed based on participants’ self-report (yes/no) of significant cybersickness side effects, such as dizziness, headache, nausea, or any other negative physical reactions.

#### Pain severity and interference

Pain severity was assessed by the 4-item Pain Severity subscale of the Brief Pain Inventory (BPI) (Cleeland and Ryan, 1994). Participants were asked to rate their pain in the last 7 days at its “worst,” “least,” “average,” and “right now” using an 11-point scale ranging from 0 = no pain to 10 = pain as bad as you can imagine. Items were averaged to obtain a total score, with higher scores representing higher pain severity (Cronbach’s *α* = 0.85). Pain interference was assessed by the 7-item Pain Interference subscale of the BPI (Cleeland and Ryan, 1994). Participants were asked to rate the degree to which, over the past week, pain has interfered with daily activities. Responses range from 0 = does not interfere to 10 = completely interferes. Items were averaged to obtain a total score, with higher scores representing higher pain interference (Cronbach’s *α* = 0.88). Pain severity and pain interference were only assessed at the pre-VR Blue timepoint. The single, “pain right now” item from the BPI was assessed pre-, mid-, and post-VR Blue. The BPI is recommended for assessing pain in clinical trials and has been readily used in cancer samples (Dworkin et al., 2008; Somers et al., 2015).

#### Tension, stress, anxiety, relaxation, and mood

Tension, stress, anxiety, mood, and relaxation levels were measured using a Visual Analog Scale (VAS) ranging from 0 = no tension, stress, anxiety, relaxation, no problem with mood to 100 = maximum tension, stress, anxiety, relaxation, extreme problem with mood. The timeframe referenced was “right now.” Tension, stress, anxiety, relaxation, and mood levels were assessed at the pre-, mid-, and post-VR Blue timepoints.

#### Pain catastrophizing

Pain catastrophizing was assessed with six items from the Coping Strategies Questionnaire (CSQ) (Keefe et al., 1989). Participants were asked about their tendencies to make negative self-statements and catastrophize when faced with pain. Responses ranged from 0 = never to 6 = always, with higher scores reflecting more pain catastrophizing. These six items were averaged to obtain a composite score (Cronbach’s *α* = 0.72). The CSQ has been previously used in patients with cancer (Somers et al., 2015, 2016).

#### Pain self-efficacy

Self-efficacy for pain management was assessed with the five-item Chronic Pain Self-Efficacy Scale (Anderson et al., 1995). Participants were asked to rate their confidence in their ability to decrease their pain and continue their daily activities. Responses ranged from 10 = very uncertain to 100 = very certain, with higher scores reflecting more pain self-efficacy. These five items were averaged to obtain a composite score (Cronbach’s *α* = 0.62). The Chronic Pain Self-Efficacy Scale has been widely used in cancer samples (Anderson et al., 1995).

#### Qualitative exit interview

At the post-VR Blue timepoint, a study staff member conducted a semi-structured, 10–15 min exit interview. Participants were asked to reflect on five categories regarding their VR experience: (1) Use of VR Technology; (2) Timing of VR Blue Session; (3) Enjoyment of VR Blue Session; (4) VR Blue Graphics; and (5) Areas for Improvement and Next Steps. Interview questions are presented in Table 5.

### Statistical analyses

Consistent with guidelines for pilot studies, we did not conduct formal statistical analyses (Kraemer et al., 2006; Eldridge et al., 2016). We computed descriptive statistics to explore pre- to post-VR Blue session changes in the main study variables (i.e., pain, tension, stress, anxiety, relaxation, and mood). Correlations were conducted to provide preliminary data on how pre- to post-VR Blue changes in two key cognitive variables (i.e., pain catastrophizing and pain self-efficacy) are related to pre- to post-VR Blue changes in the main study variables. Qualitative exit interview data were coded using open coding and memoing by two members of the study team to generate repeated concepts. Results were categorized into major themes through selective coding methods and methods from Applied Thematic Analysis (Guest et al., 2011) were used to evaluate the qualitative interview data.

## Results

### Participant characteristics

Demographic and medical characteristics are reported in Tables 1 and 2, respectively. The average age of participants enrolled in this study was 56.55 (SD = 10.73). The sample was 70% male (*n* = 14). One-quarter of the sample identified as Black/African-American (*n* = 4). The majority of participants were non-Hispanic (90%). Most participants reported a college education, with 40% obtaining a graduate degree.

### Feasibility

#### Accrual

**Fig 4.**
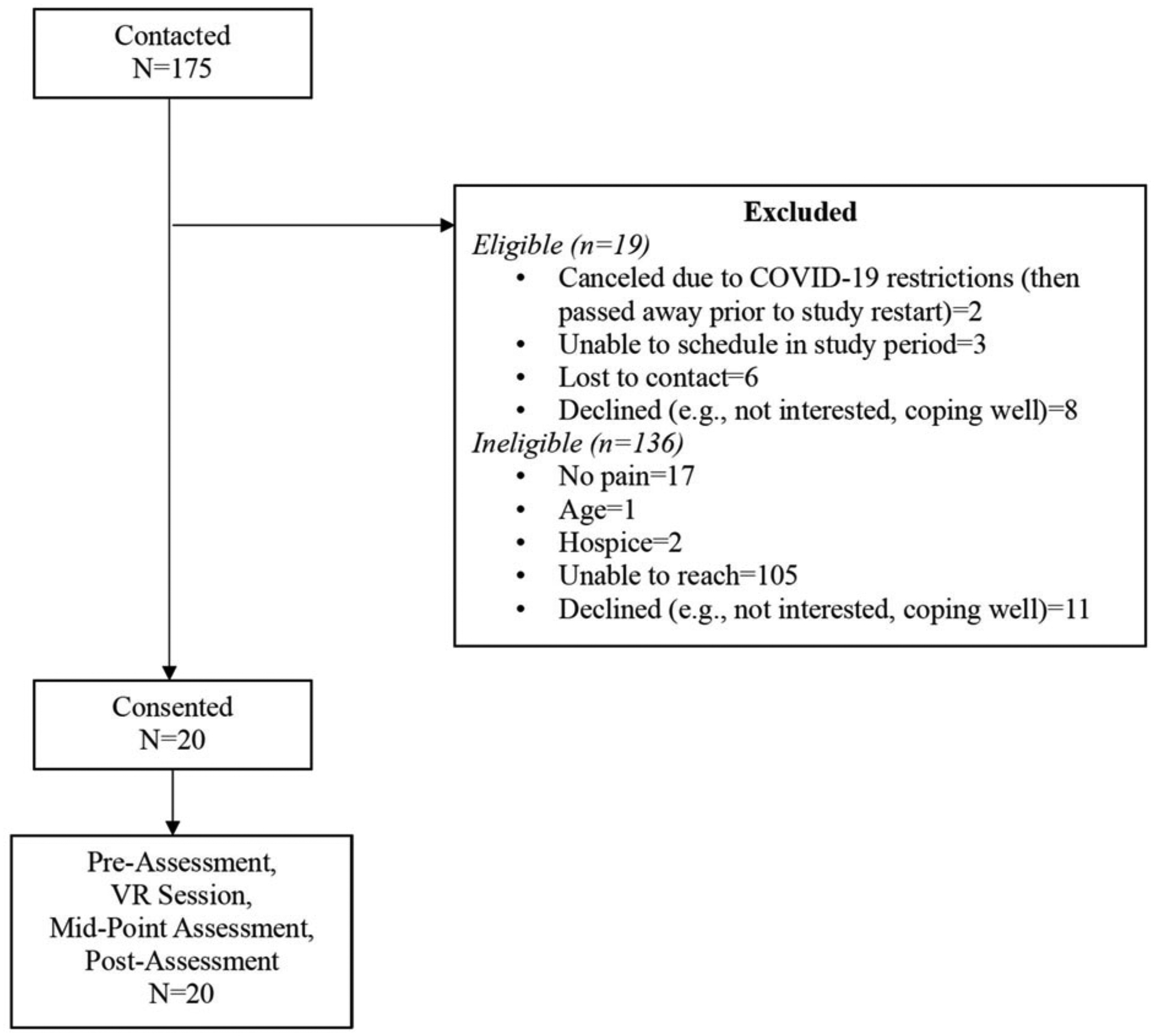
CONSORT.

Of the 175 patients who met inclusion criteria and were contacted for a telephone screening, 91 were unreachable and 21 were ineligible (see Figure 4 for CONSORT diagram). We enrolled 20 participants in a total of 15 months. Safety restrictions put in place by the Duke University Health System because of COVID-19-interrupted recruitment for 9 months. Thus, the total *active* recruitment time across the 15-month period was 6 months. This was aligned with our feasibility benchmark to recruit 20 participants in 6 months.

#### Adherence

All participants (100%) completed the single 30-min VR Blue session. This was above our 80% completion rate to indicate feasibility.

#### Data completion

There was 100% data collected at the pre-, mid-, and post-VR Blue assessments. This was above our feasibility benchmark of 80% completed data across all assessment timepoints.

#### Acceptability

Participants found the VR Blue protocol to be highly acceptable with a mean satisfaction rating of 3.30 out of 4.0 (SD = 0.41). This was above our 80% satisfaction benchmark (*M* = 3.2/4.0) to indicate feasibility.

#### Safety

All participants (100%) completed the VR Blue session without self-report of significant side effects (e.g., dizziness, headache, and nausea). One participant noted that if he moved his head too quickly, he noticed mild dizziness, but otherwise did not experience dizziness and said he was not bothered by this. This met our feasibility benchmark of 80% completion without significant side effects.

### Pre- to post-VR Blue changes in main study variables

Means and standard deviations for main study variables are reported in Table 3. From pre- to post-VR Blue, pain “right now” decreased by 58.93%. From pre- to post-VR Blue, tension decreased by 74.33%. From pre- to post-VR Blue, stress decreased by 68.40%. From pre- to post-VR Blue, anxiety decreased by 65.22%. From pre- to post-VR Blue, relaxation increased by 37.78%. From pre- to post-VR Blue, mood level improved by 70.20%. In Table 4, we present pre- to post-VR Blue changes in key cognitive variables (i.e., pain catastrophizing and pain self-efficacy) in relation to the main study variables. Change in pain catastrophizing was significantly correlated with change in relaxation (*r* = −0.455, *p* < 0.05). Although no other correlations reached statistical significance, the following were in the expected direction: (1) change in pain catastrophizing and changes in pain, tension, stress, and anxiety and (2) change in pain self-efficacy and changes in stress, anxiety, and relaxation. These results should be interpreted cautiously due to the small sample size.

### Qualitative exit interview

Data from the exit interview is detailed in Table 5.

#### Ease of VR technology

The majority of participants reported the VR headset was easy to use (*n* = 19) and comfortable (*n* = 17). Most participants reported they felt immersed in the VR Blue scenery (*n* = 17). Several participants reported they were open to engaging in VR in *either* the medical setting *or* at home (*n* = 4), but the majority preferred their home (*n* = 13).

#### Timing of VR the Blue session

Approximately half of the participants stated the length of the VR Blue session was appropriate, while the other half reported they would like it to be longer. Most participants (*n* = 11) said they would use VR Blue multiple times per week. Many (*n* = 12) stated they would use VR Blue for pain and symptom management at the time of their cancer treatment appointments. Participants stated they were most interested in using VR Blue when pain, anxiety, and/or depression are high (*n* = 13).

#### Enjoyment of the VR Blue session

Nineteen participants stated they enjoyed the VR Blue session. One participant felt “neutral” about the experience.

#### VR Blue session graphics

All participants reported they enjoyed the VR Blue scenery. Many participants (*n* = 13) noted if they had regular access to VR, they would like the option to change scenic genres (e.g., outer space and observation of wildlife).

#### Improvement/next steps

Most participants (*n* = 12) were open to having caregivers involved in the VR Blue session and thought caregivers would be amenable to participating. Recommended improvements included a cordless headset and a higher resolution picture.

## Discussion

VR interventions are an emerging tool for managing bothersome symptoms such as pain and distress, but there is little work examining the utility of these programs for *persistent* pain in the cancer setting. Advanced colorectal cancer patients that suffer from high symptom burden and distress may particularly benefit from an immersive, non-pharmacological approach such as VR. This is the first study to assess the initial feasibility, acceptability, safety, and impact of a single, 30-min VR intervention on the clinical pain experience of advanced colorectal cancer patients.

We found that the VR Blue protocol was highly feasible. From the 84 patients that were eligible, 20 were recruited and enrolled in the study in a 6-month timeframe. All enrolled participants were willing and able to complete the VR Blue session. Likewise, there was 100% data collected for all participants at all assessment timepoints. These data meet all *a priori* feasibility benchmarks and suggest that patients with advanced colorectal cancer were attracted to the VR protocol and willing to enroll in the pilot study. Furthermore, once consented, participants completed all study activities as outlined in the protocol. This indicates the recruitment, retention, and assessment procedures used in this pilot study are appropriate, and ready for further testing in larger-scale, randomized efficacy trials.

Our results also show excellent acceptability of the VR Blue protocol, with the mean rating reflecting “high” acceptability. This is strong preliminary support for the appeal of a VR intervention in this patient population. Importantly, our safety benchmark was met as well, with all participants reporting no significant side effects. This was the first pilot study to test a VR intervention in a sample of advanced colorectal cancer patients; thus, it is noteworthy that the protocol was both acceptable and safe based on *a priori* benchmarks. These results strengthen the argument for moving to the next step of the NIH Stage Model to conduct a large, randomized efficacy trial.

Prior to efficacy testing, further intervention development and refinement may be warranted. Results from our qualitative interview are a useful complement to questionnaire data, and offer insight into participants’ preferences, thoughts, and feelings regarding the current VR Blue protocol. Overall, participants described the VR technology as easy to use and comfortable, and described the VR Blue session as highly immersive and enjoyable. Most participants reported the length of the VR Blue session felt appropriate, and some stated they would enjoy a longer session. Also, most participants reported they would use VR Blue multiple times per week (if not every day) and indicated a preference for use in the home. Several participants shared they were most interested in using it when symptom (e.g., pain and anxiety) levels are high. Participant suggestions for improving the protocol included recommendations for other scenery options and a cordless headset. Another area for further consideration is the inclusion of caregivers in the VR Blue session. During the qualitative interviews, most participants indicated they were open to having caregivers involved. There is a known dyadic relationship between a cancer patient and the caregiver, such that the way a patient copes with symptoms (i.e., pain) can influence how a caregiver copes with their own caregiving burden, and vice versa (Hamidou et al., 2018; Lingens et al., 2021). Involving caregivers in the VR experience might align patient and caregiver coping responses, ultimately enhancing the impact of the VR intervention and improving the quality of life for both the patient and the caregiver. Still, questions remain regarding how and when caregivers should be included in the VR Blue session. Taken together, this feedback highlights several areas of potential intervention refinement that could be explored through additional pilot work before progressing through the NIH Stage Model (Onken et al., 2014).

As this was a pilot study primarily focused on assessing feasibility, acceptability, and safety, we did not conduct formal statistical analyses. However, descriptive statistics showed the pre- to post-VR Blue changes were in the hypothesized direction for all main study variables. Particularly robust improvements were observed for pain, tension, stress, anxiety, and mood, which evidenced changes of 59% or greater. All pre-post changes were above our *a priori* MCID of 30%, suggesting that these outcomes are meaningfully affected by the VR intervention. Furthermore, the correlation between pre- to post-VR Blue change in relaxation and change in the secondary outcome of pain catastrophizing was significant. This finding may be particularly important as patients with pain that have high levels of pain catastrophizing are much more likely to experience persistent pain and other negative pain-related outcomes (Quartana et al., 2009; Somers et al., 2012; Syrjala et al., 2014). VR may uniquely lead to improvements in pain catastrophizing ultimately leading to better overall outcomes for patients with pain. This potential mechanism of change should be explored further in future work.

Some limitations should be noted. First, due to the pilot nature of this work, our sample size was small; a larger sample will provide adequate power for testing significant differences. Second, we focused this early work on colorectal cancer patients; future work should include other disease groups. Our pilot study had several strengths. We used a novel mode of intervention with an under-studied cancer population with unique symptom management needs. Our design was comprehensive in its use of quantitative and qualitative methods, which yielded important data for refining this VR intervention. Finally, it is notable that despite disruptions to recruitment due to COVID-19, the accrual target was met, suggesting the appeal of this VR intervention.

## Conclusion

Non-pharmacological pain management options are needed for advanced colorectal cancer patients who suffer from high symptom burden. VR is an engaging experience that can be conveniently used in a variety of settings and may have the potential to reduce pain and pain-related symptoms to ultimately improve patient outcomes. However, the first step in assessing the utility of VR interventions for advanced colorectal cancer patients is examining the feasibility, acceptability, and safety of such a protocol.

Quantitative and qualitative results from this pilot study offer strong preliminary evidence that this single, 30-min VR protocol is feasible, acceptable, and safe for patients with advanced colorectal cancer. Data from semi-structured interviews highlight possibilities for next steps in this program of research, including testing multi-session and home-based VR protocols, use of VR in the medical setting during cancer treatments/procedures, and use of VR in the patient’s home with caregivers and/or family members. Another interesting avenue for future work is the use of VR to enhance existing cognitive-behavioral protocols for pain and symptom management. For example, VR could be used to deepen the experience of relaxation exercises (e.g., imagery) and/or serve as a scheduled pleasant activity. Ultimately, larger, randomized clinical trials with a control condition are needed to examine the efficacy of VR-based interventions for patients with advanced colorectal cancer and pain. This research could lead to VR-based interventions that could benefit other advanced-stage cancer populations and/or other palliative care populations who are experiencing persistent pain and distress.

## Figures and Tables

**Table 1.** Participant demographic characteristics (*N* = 20)

	*N* (%)	*M*	SD	Range
Age (years)		56.66	10.73	32–80
Gender				
Male	14 (70%)			
Female	6 (30%)			
Race				
White	15 (75%)			
Black or African American	4 (20%)			
Native Hawaiian or Pacific Islander	1 (5%)			
Ethnicity				
Non-Hispanic or Latino	18 (90%)			
Hispanic or Latino of any race				
Declined to report				
Unknown	1 (5%)			
Other	1 (5%)			
Education				
Less than HS	1 (5%)			
HS diploma	3 (15%)			
Some college	4 (20%)			
Bachelor’s degree	4 (20%)			
Graduate degree	8 (40%)			
Marital Status				
Never married	2 (10%)			
Married	16 (80%)			
Widowed	2 (10%)			

*M*, mean; SD, standard deviation; HS, High school.

**Table 2.** Participant medical characteristics (*N* = 20)

	*N* (%)	*M*	SD	Range
First cancer or recurrence				
First cancer	15 (75%)			
Recurrence	5 (25%)			
Time since diagnosis (months)		40.70	40.82	2.33–157.17
Chemotherapy				
Yes	20 (100%)			
No				
Radiation				
Yes	9 (45%)			
No	11 (55%)			
Surgery				
Yes	15 (75%)			
No	5 (25%)			
Pill or anti-cancer drug				
Yes	4 (20%)			
No	16 (80%)			
Immunotherapy				
Yes	3 (15%)			
No	17 (85%)			
Days with pain medication		3.25	3.21	0–7
OTC pain medication				
Yes	11 (55%)			
No	9 (45%)			
Opioid medication				
Yes	10 (50%)			
No	10 (50%)			
Anticonvulsants				
Yes	1 (5%)			
No	19 (95%)			
Cannabis and/or CBD				
Yes	1 (5%)			
No	19 (95%)			
Anti-anxiety medication				
Yes	1 (5%)			
No	19 (95%)			

*M*, mean; SD, standard deviation; OTC, over the counter; CBD, cannabidiol.

**Table 3.** Means (standard deviations) for main study variables: pre-, mid-, and post-VR Blue

	Pre-VR Blue	Mid-VR Blue	Post-VR Blue
	M (SD)	M (SD)	M (SD)
BPI: pain severity	3.15 (1.46)		
Worst	5.60 (2.54)		
Right Now	2.80 (1.44)	1.35 (1.23)	1.15 (1.14)
Least	1.05 (1.05)		
Average	3.15 (1.66)		
BPI: pain interference	2.86 (2.07)		
General activity	3.65 (3.00)		
Mood	2.55 (2.50)		
Walking ability	3.25 (2.99)		
Normal work	3.70 (2.62)		
Relations with others	1.30 (1.63)		
Sleep	3.20 (3.46)		
Enjoyment of life	2.35 (2.52)		
Visual Analog Scales (0–100)			
Expected Improvement	56.05 (20.21)		
Tension Right Now	30.00 (24.39)	10.75 (13.31)	7.70 (12.70)
Stress Right Now	24.05 (23.79)	5.25 (11.06)	7.60 (13.59)
Anxiety Right Now	19.55 (23.06)	6.25 (12.23)	6.80 (12.56)
Relaxation Right Now	62.20 (21.39)	83.20 (16.59)	85.70 (17.34)
Mood Right Now	12.75 (16.42)	3.30 (9.20)	3.80 (11.31)
Enjoyment			87.45 (12.46)
Pain Self-Efficacy	59.90 (18.43)		72.60 (16.49)
Pain Catastrophizing	1.10 (0.92)		0.88 (0.89)
Client Satisfaction Questionnaire			3.30 (0.41)
Quality of VR session			3.65 (0.59)
VR gave helpful skills			3.35 (0.67)
Extent VR met your needs			2.80 (0.77)
Recommend VR			3.90 (0.31)
Satisfied with help			3.65 (0.75)
Helped deal with pain			3.20 (0.70)
Overall satisfaction			3.60 (0.60)
Consider using for pain/tension			3.75 (0.44)
Helped understand pain			2.75 (0.64)
Gave coping skills for pain/tension			2.35 (0.75)

*N*, 20; *M*, mean; SD, standard deviation; VR, virtual reality; BPI, Brief Pain Inventory.

**Table 4.** Correlations of change scores for key cognitive variables and main study variables

Variable	Δ Pain	Δ Stress	Δ Anxiety	Δ Tension	Δ Mood	Δ Relaxation
Δ PS	0.004	−0.014	−0.370	0.187	0.233	0.281
Δ PC	0.306	0.398	0.342	0.327	−0.119	−0.455*

Δ denotes change; PC, pain catastrophizing; PS, pain self-efficacy; change scores for main study variables, pre-VR minus post-VR.

* *p* < 0.05.

**Table 5.** Qualitative exit interview data

Theme	Questions	Relevant participant feedback
Use of VR Technology	Was the VR technology (i.e., headset) comfortable (e.g., fit and feel)? Was the VR technology (i.e., headset, screen, and controller) easy to use? Did you have a sense of immersion in VR Blue? Would you prefer to do this in your home or in the medical center setting?	Headset was easy to use (*n* = 19) and comfortable (*n* = 17); wearing VR headset with eyeglasses was not difficult (*n* = 3) Some reported headset was slightly heavy (*n* = 4) Cord connecting the headset to the computer was distracting (*n* = 3) Gap where participants can see outside the VR headset (*n* = 2) Felt immersed (*n* = 17) Prefer doing VR in home (*n* = 13); medical setting (*n* = 3); either (*n* = 4)
Timing of VR Blue	Did the length of the VR Blue session (30 min) feel appropriate? How often would you use VR Blue? What time of day would you use VR Blue? When along the cancer experience would you use VR Blue?	Length felt appropriate (*n* = 13) Would like the VR Blue session to be longer (*n* = 7); would like the third underwater ocean scene to be shorter (*n* = 2) Would use VR Blue everyday (*n* = 6) Would use VR multiple times per week (*n* = 11) Evening (*n* = 10); afternoon or evening (*n* = 6); afternoon only (*n* = 3); anytime (*n* = 1) Would use VR Blue at the time of cancer diagnosis (*n* = 6), in alignment with other cancer treatments (*n* = 12), later during cancer experience (*n* = 4), and/or during times of particularly high pain, anxiety, or depression (*n* = 7)
Enjoyment	Did you enjoy VR Blue?	Enjoyed (*n* = 19) Neutral (*n* = 1)
VR Blue Graphics	What was your reaction to the underwear ocean scenes? Would you appreciate the option to use different types of scenery? If you use VR Blue regularly, what type of scenery would you like to use?	Enjoyed at least one of the underwater ocean scenes (*n* = 20) Preferred same scenery/theme (*n* = 7); open to different scenery (*n* = 13) Suggestions for different scenery included: outer space, forest, mountains, caves, observation of wildlife, time-lapse videos of travel
Areas for Improvement/Next Steps	What would make VR protocol better? Do you think your partner/caregiver would be interested/willing to be involved in your use of VR Blue? Would you want your partner/caregiver involved in your use of VR Blue?	Recommendations for improvement included cordless headset and higher resolution picture Most felt that partners/caregivers would be interested and willing to be involved in the use of VR Blue (*n* = 12) There were no strong opinions regarding advantages/disadvantages of having partners/caregivers involved in the use of VR Blue
